# Supplementary figures and images for: Natural variants in SARS-CoV-2 Spike protein pinpoint structural and functional hotspots with implications for prophylaxis and therapeutic strategies
Source: Sci Rep. 2021 Jun 23;11:13120. doi: 10.1038/s41598-021-92641-x (PMC8222349; doi:10.1038/s41598-021-92641-x)

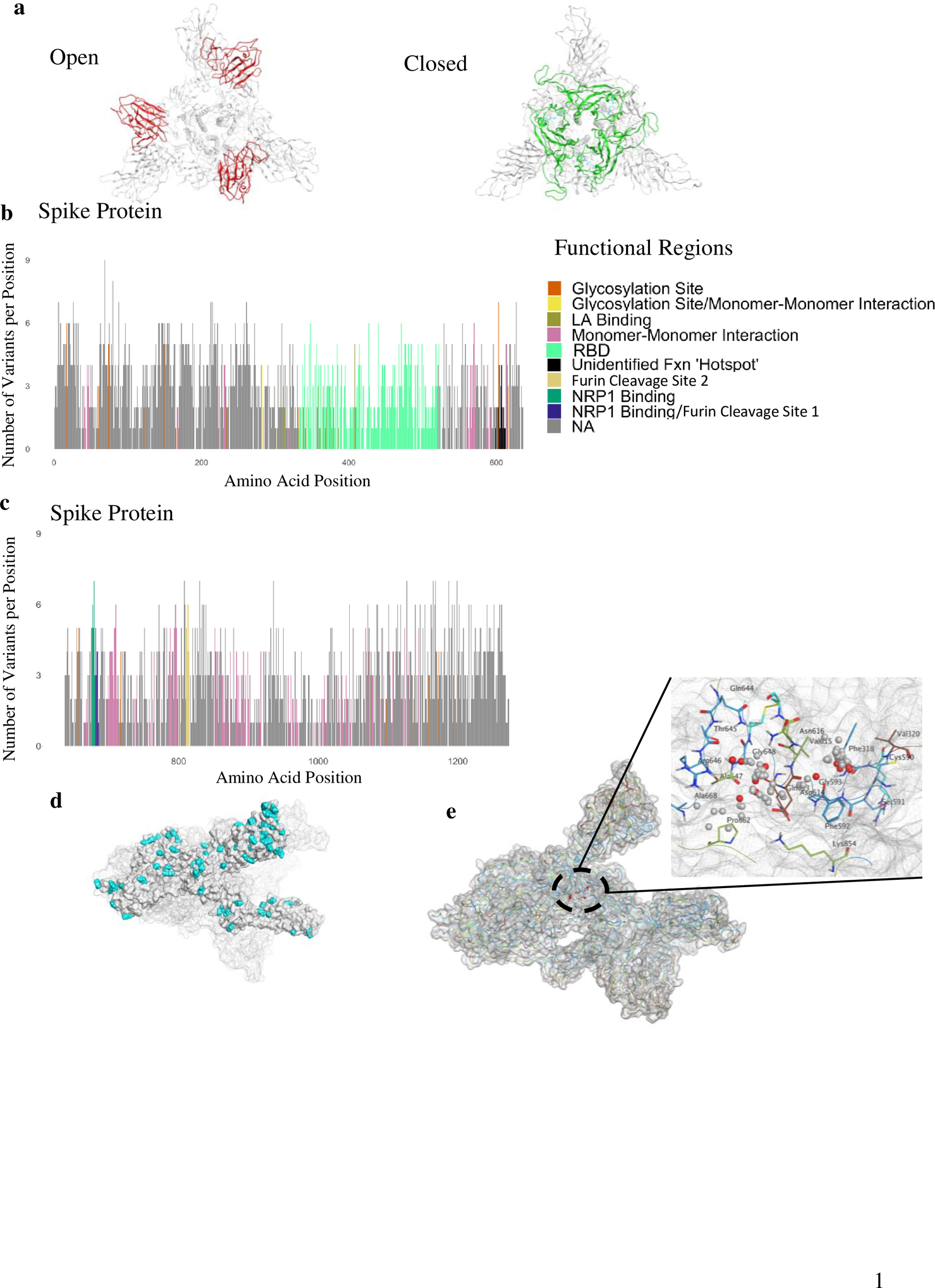

Supplement: Supplementary file 2 — Supplementary Information 2. [file 41598_2021_92641_MOESM2_ESM.zip › Supplemental Figure 1 042621.tif]
