# Supplementary material for: Natural variants in SARS-CoV-2 Spike protein pinpoint structural and functional hotspots with implications for prophylaxis and therapeutic strategies
Source: Sci Rep. 2021 Jun 23;11:13120. doi: 10.1038/s41598-021-92641-x (PMC8222349; doi:10.1038/s41598-021-92641-x)
Supplement: Supplementary file 5 — Supplementary Information 5. [file 41598_2021_92641_MOESM5_ESM.docx]

**Supplementary figure 1.** Variant and invariant regions in SARS-CoV-2 Spike (S) protein. **a)** SARS-CoV-2 S protein trimer showing the receptor-binding domain (RBD) in the open conformation (red; PDB ID: 7A98) and the closed conformation (green; PDB ID: 6ZB5). Viewed from the top. **b & c**) Regions in the sequence (1-636: **b**; 637-1273:**c**) of the S protein are color-coded according to function. **d)** New potential glycosylation sites (cyan) on S protein in circulating variants. **e)** Potential ligand binding site in the hotspot (aa 541-612). Residues that form the potential binding site are highlighted and colored according to the number of variants per position, as in figures 1-4. Red (hydrophilic) and white (hydrophobic) spheres represent dummy atoms in the potential binding site.
